# Bioinformatic approach to explain how Mg from seawater may be incorporated into coral skeletons

**DOI:** 10.1098/rsos.232011

**Published:** 2025-01-22

**Authors:** Tomoko Bell, Akira Iguchi, Yoshikazu Ohno, Kazuhiko Sakai, Yusuke Yokoyama

**Affiliations:** ^1^ Division of Science and Mathematics, Newman University, Wichita, KS 67213, USA; ^2^ Geological Survey of Japan, National Institute of Advanced Industrial Science and Technology (AIST), Tsukuba 305-8567, Japan; ^3^ Research laboratory on environmentally conscious developments and technologies, National Institute of Advanced Industrial Science and Technology (AIST), Tsukuba 305-8567, Japan; ^4^ School of Marine Biosciences, Kitasato University, Kanagawa 252-0373, Japan; ^5^ Sesoko Station, Tropical Biosphere Research Center, University of the Ryukyus, Okinawa 905-0227, Japan; ^6^ Atmosphere and Ocean Research Institute, The University of Tokyo, Kashiwa 275-8564, Japan; ^7^ Department of Earth and Planetary Science, The University of Tokyo, Bunkyo 113-0033, Japan

**Keywords:** coral Mg transporter, evolution, biological effects, coral skeletons, geochemical proxy

## Abstract

Corals have been used as geochemical proxies since the 1970s, playing a prominent role in paleoceanography. However, it has not been well elucidated how aqueous ions sourced from seawater are transported and precipitated in coral skeletons. There are limited foundational methods to differentiate and quantify biogenic and abiogenic effects during skeletal formation. Especially, Mg in coral skeletons show individual variations suggesting large biogenic effects. Here, we evaluated biological complexity by investigating how coral genes evolved over geologic time scales. We focused on Mg transporter and analysed five species from genus *Acropora* and three species from genus *Porites*. Mg transporter of *Acropora digitifera*, *Acropora hyacinthus*, *Acropora millepora* and *Porites australiensis* showed higher similarity to Mg transporter of vertebrates and were reported to appear on Earth during the Pleistocene. On the other hand, *Acropora palmata*, *Acropora tenui*s and *Porites astreoides* showed lower or no similarity to vertebrates, and they were reported to appear on Earth before the Pleistocene. We suggest such evolutional records can be evidence to demonstrate biological complexity of Mg transport from seawater. This might explain that Mg transport is subject to evolution and why Mg incorporated in coral skeletons tends to show strong biogenic effects compared with other elements.

## 1. Introduction

Coral skeletal elements from both modern and fossil samples have been used as geochemical proxies since the 1970s and play prominent roles in elucidating past environments (e.g. sea temperature and pH; [1,2]). However, some elements are reported to record environments less accurately than others. For example, Mg is one of the essential elements incorporated into the coral skeletons and widely studied and analysed in paleoclimatology (e.g. *Porites* sp. and *Acropora* sp.) as a tool to reconstruct sea temperature [3–8]. Interestingly, most of the studies concluded that coral skeletal Mg was not an accurate indicator to reconstruct past temperature [9,10]. Although Mg is a significant element of focus in geochemistry and paleoceanography, its mechanism to be incorporated into coral skeletons and the reasons why Mg is not an ideal temperature proxy have not been elucidated. Thus, further research of coral skeletal Mg is needed.

In coral molecular biology, the transportation and regulation of dissolved inorganic carbon, Ca^2+^ and H^+^ related to calcification have been intensively studied. Thus, functions of enzymes such as carbonic anhydrase, bicarbonate transporters, calcium channels, CaATPase and Na^+^–H^+^ exchangers have been reported [11]. Also, using a cutting-edge imaging method, the calcifying mechanism is becoming clear [12,13]. Furthermore, amorphous calcium carbonate might play a significant role in the formation of aragonite via vesicle transport pathways in the coral and other biomineralizing organisms’ calcifying tissue [14,15]. However, even with the great progress of current studies, the mechanisms of how aqueous ions sourced from seawater are transported into and precipitated in aragonite coral skeletons are not fully understood, specifically how Mg-related transporters affect the mineral composition in coral skeletons has not been reported.

This knowledge gap between molecular biology and geochemistry/paleoceanography can be summarized as the following. Foundational methods to differentiate and quantify biogenic and abiogenic effects during skeletal formation are still under development, hampering the application of corals as fully reliable environmental proxies. To address this issue, the use of a coral genome database to separate biogenic from abiogenic effects of skeletal formation has been suggested in 2018 [16]. This study evaluated the magnitude of biogenic effects by counting the number of genes related to each skeletal element (e.g. Na, Mg, Ca, Sr, U and Li) based on the assumption that biological complexity is partially related to an increase in number of genes [17]. In other words, the number of genes is correlated with the complexity of specific systems and can be useful to compare among interspecies or different systems within one species. For example, within well-studied model organisms, birds have 100−650 genes for olfactory receptors, whereas zebra fish, humans and mice have 100, 339 and 1000 genes, respectively [18]. In this case, we can conclude that mice have the most complex olfactory system. Another example is that corals have specific transporters related to Na, Mg and Ca, while no transporters were found exclusively for Sr, U and Li. That is to say, corals have more complex systems to transport, Na, Mg and Ca compared with Sr, U and Li [16].

Although such an approach provides significant insights into specific aspects of biological effects and complexity, relying solely on the number of genes must be performed with caution. Possible risks of this approach include: (i) the number of genes varies depending on datasets and/or cut-off value criteria; (ii) the number of expressed genes can change depending on experimental and/or environmental conditions; and (iii) the effects of non-coding DNA regions are neglected. To address these uncertainties, we attempt to evaluate biological effects and complexity by investigating how coral genes have been conserved and/or evolved over geologic (million-year) time scales. We applied a simple idea to bridge the gap between molecular biology and geochemistry/paleoceanography. That is to say, biologically complex systems are subject to evolution, while abiogenic processes are not. This concept was inspired by the fact that there were significant similarities in gene sequences between corals and humans. The major components of the human Ca ion trafficking system were identified in coral genomic data [19].

In this study, to quantify biological effects and complexity in coral skeleton formation, we focus on Mg ions and coral Mg transporters using a bioinformatics approach. As previously stated, the mechanism of Mg transport into coral skeletons is significantly understudied compared with Ca, although Mg transporters have been reported to impact biomineralization using other animals, such as humans and mice [20]. In addition to counting the number of Mg transporters, we investigated the evolution of coral Mg transporters by comparing protein sequences from five different *Acropora* sp., three different *Porites* sp. and vertebrate animals (amphibian, fish, reptile, bird, orangutan and humans) using sequence similarity search. Then, we interpreted the results from our bioinformatics approach to assess biological complexity, possible biological effects on coral skeletal proxies in paleoceanography.

## 2. Results

### 2.1. Mg transporter of *Acropora digitifera*: homologue of non-imprinted in Prader–Willi/Angelman syndrome family

We discovered that aug_v2a.24016.t1 in *Acropora digitifera* v. OIST_v.1.1 was the only sequence described as an Mg transporter in the database. This sequence was statistically similar (e-value = 0 and sequence identity = 52%) to the National Center for Biotechnology Information (NCBI) Reference Sequence: XP_004068355.1, Mg transporter non-imprinted in Prader–Willi/Angelman syndrome (NIPA2) isoform X2 [*Oryzias latipes*, Japanese rice fish]. In the newer version of this dataset (OIST_v.2), there were three sequences described as Mg transporters, and the best hit was adig_s0124.g5.t1. This sequence was also statistically similar (e-value = 0, and sequence identity = 64%) to the NCBI Reference Sequence splQ5R7Q3.1, Mg transporter NIPA2 [*Pongo abelii*, orangutan]. Thus, both versions of the database suggest the Mg transporter of *A. digitifera* is highly similar (>52%) to the transporter of vertebrates. NIPA is known to be associated with early endosomes and the cell surface in a variety of neuronal and epithelial cells [21].

### 2.2. Mg transporter NIPA and NIPA-like proteins among different coral species that appeared during Pleistocene and Pre-Pleistocene

A basic local alignment search tool (BLAST) search was conducted using Mg transporter of *A. digitifera* from the result above (adig_s0124.g5.t1, OIST-v2) as the query sequence and the protein databases of *A. digitifera*, *Acropora hyacinthus*, *Acropora millepora*, *Acropora palmata*, *Acropora tenuis, Porites australiensis, Porites astreoides* and *Porites lobata* from Reefgenomics as subject sequences. Three of the *Acropora* databases (*A. digitifera*, *A. hyacinthus* and *A. millepora*) possessed two statistically similar Mg transporters (e-values < 1 × 10^−10^): NIPA and/or NIPA-like (NIPAL). On the other hand, *A. tenuis* had only one Mg transporter, and *A. palmata* had no hits. Two *Porites* databases (*P. australiensis* and *P. lobata*) had two statistically similar Mg transporters (e-values < 1 × 10^−10^), while *P. astreoides* had only Mg transporter (table 1).

**Table 1.** Results of sequence similarity search among *Acropora* sp., *Porites* sp., *Oryzias latipes* (rice fish), *Pongo abelii* (orangutan), and human. Reefgeneomics sequences were extracted using seqkit (https://bioinf.shenwei.me/seqkit/) and annotated using NCBI BLASTP (BLAST+ 2.15.0).

reefgenomics (Gene ID)	OIST genomic projects v2	NCBI	NCBI	NCBI
	*Acropora digitifera* Mg transporter (NIPA)	*Pongo abelii* Mg transporter (NIPA)	*Oryzias latipes* Mg transporter (NIPA)	*Homo sapien*s Mg transporter (NIPA)
	adig_s0124.g5.t1	Q5R7Q3.1	XP_004068355.1,	NP_001171818.1
	identity (%) (e-value)	identity (%) (e-value)	identity (%) (e-value)	identity (%) (e-value)
*Acropora digitifera*				
2046 (NIPA)	99 (0)	64 (2 × 10^−140^)	58 (3 × 10^−141^)	64 (1.71 × 10^−140^)
13 221 (NIPAL)	33 (7 × 10^−39^)	31 (3 × 10^−39^)	29 (7 × 10^−36^)	31 (3.41 × 10^−39^*)*
*Acropora millepora*				
23 156 (NIPA)	99 (0)	64 (3 × 10^−140^)	58 (2 × 10^−140^)	64 (2.65 × 10^−140^)
12 886 (NIPAL)	35 (2 × 10^−36^)	31 (4 × 10^−36^)	28 (3 × 10^−32^)	31(3.72 × 10^−36^)
*Acropora hyacinthus*				
6007 (NIPA)	96 (1 × 10^−97^)	60 (9 × 10^−44^)	65 (2 × 10^−47^)	60 (9.09 × 10^−44^)
3879 (NIPAL)	29 (9 × 10^−18^)	28 (3 × 10^−19^)	25 (2 × 10^−15^)	28 (3.41 × 10^−19^)
*Acropora tenuis*				
8750 (NIPAL)	38 (2 × 10^−22^)	37 (1 × 10^−21^)	34 (4 × 10^−19^)	37 (1 × 10^−21^)
*Acropora palmata*				
no hits	no hits	no hits	no hits	no hits
				
*Porites australiensis*				
5842 (NIPA)	80 (0)	65 (2 × 10^−144^)	60 (1 × 10^−145^)	65 (2 × 10^−144^)
5886 (NIPAL)	32 (1 × 10^−39^)	29 (5 × 10^−36^)	28 (6 × 10^−33^)	29 (5 × 10^−36^)
*Porites lobata*				
16 155 (NIPA)	79 (0)	64 (2 × 10^−143^)	60 (3 × 10^−146^)	64 (2 × 10^−143^)
15 472 (NIPAL)	32 (6 × 10^−37^)	29 (3 × 10^−36^)	28 (4 × 10^−33^)	29 (3 × 10^−36^)
*Porites astreoides*				
1808 (NIPA)	85 (3 × 10^−33^)	69 (3 × 10^−25^)	62 (5 × 10^−26^)	69 (3 × 10^−25^)

Four coral species (*A. digitifera*, *A. hyacinthus*, *A. millepora* and *P. australiensis*) possessed species ages of less than 2.58 Ma (start of Pleistocene) in the Coral Trait Database, whereas the ages of the other three species (*A. palmata*, *A. tenuis* and *P. astreoides*) were greater than 2.58 Ma (table 2). Hereafter, we refer to the four species that appeared on Earth during the Pleistocene as the ‘Pleistocene group’, and the other three species as the ‘pre-Pleistocene group’. Combining these results from Reefgenomics and Coral Trait Database, interestingly, the Pleistocene group have both NIPA and NIPAL in the database. It should be noted that *P. lobata* was not listed in the database, and the data of its species age were not available.

**Table 2.** Species age phylogeny data (Ma) of five *Acropora* sp. and three *Porites* sp. from Coral Trait database (Madin *et al*., 2016; https://coraltraits.org/).

coral trait database	species age phylogeny data (Ma)
*Acropora digitifera*	0.72
*Acropora millepora*	0.89
*Acropora hyacinthus*	0.58
*Porites australiensis*	1.47
*Acropora palmata*	3.24
*Acropora tenuis*	3.46
*Porites astreoides*	9.89
*Porites lobata*	Not available

### 2.3. Pleistocene group shows high similarity with fish, orangutan and human

We compared the sequence similarity between the results for the five *Acropora* and three *Porites* species (left column in table 1), and *O. latipes* (rice fish) XP_004068355.1_Mg transporter NIPA2 isoform X2 and *P. abelii* (orangutan) XP_004068355.1_ Mg transporter NIPA2, which were the descriptions for the *A. digitifera* Mg transporter in OIST_v.1.1. and v2, respectively. In addition, the sequence similarity between the results from all the coral species and the total of 202 human Mg-transporter sequences from NCBI databases were compared. The Pleistocene group carried NIPA and NIPAL, and their NIPA displayed high similarity (e-values < 1 × 10^−40^) with the rice fish, orangutan and human NIPA, whereas no such pattern was apparent for the pre-Pleistocene group (table 1).

### 2.4. Vertebrate Mg transporter NIPA: possible pre-Pleistocene origin

**Figure 1.**
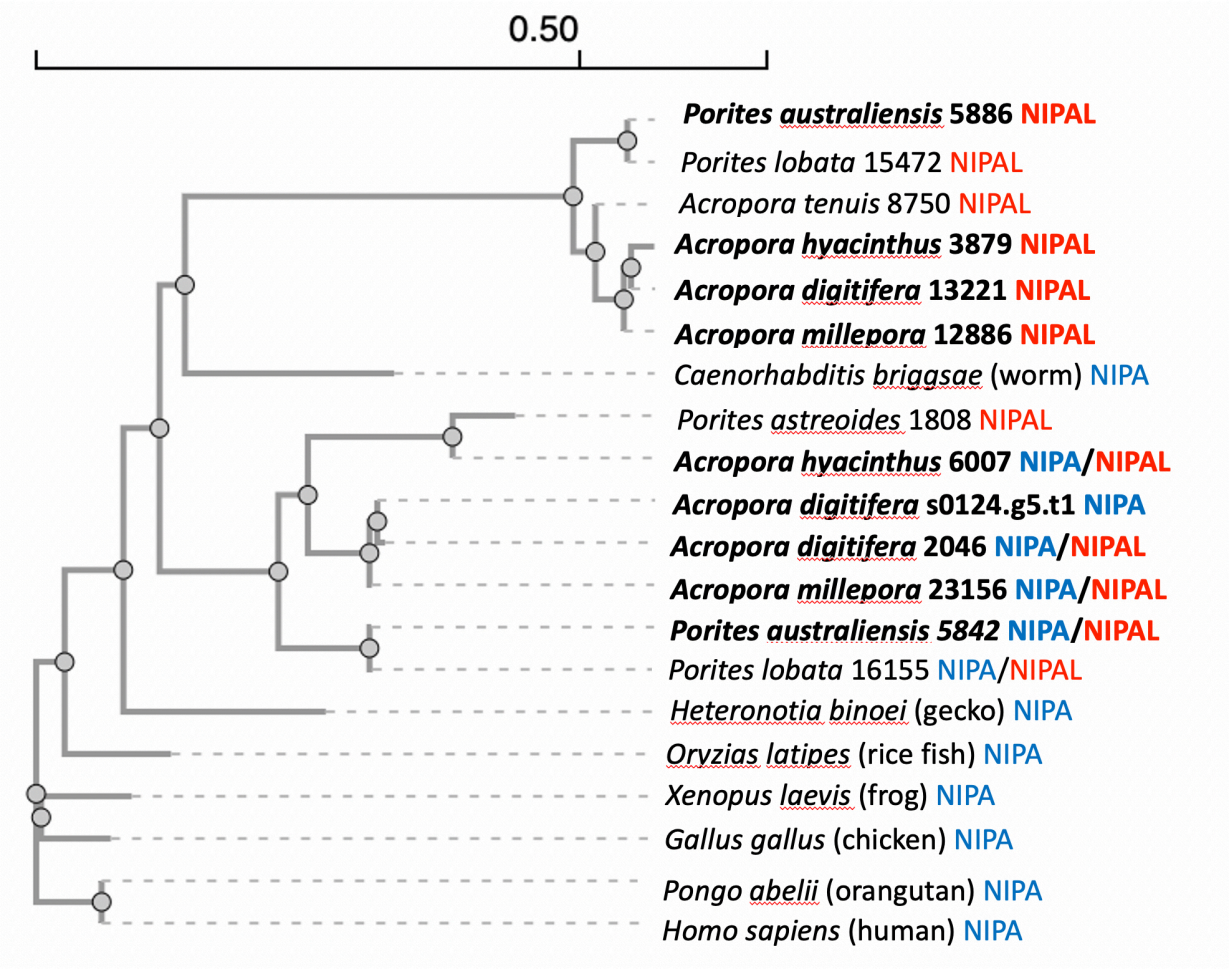
Phylogenic tree of Mg transporters. Coral species in bold are reported to appear on Earth during Pleistocene (<2.58 Ma) based on Coral Trait database (https://www.coraltraits.org/). They show higher similarities to vertebrate Mg transporter NIPA. Also, they have both NIPA and NIPAL proteins and showed the possible evolution from NIPAL (NIPA-like) to NIPA. It should be noted that *P. lobata* was not listed in the Coral Trait database, and the data of species age was not available. Scale bar represents amino acid variation for the length.

We constructed a phylogenic tree from 20 protein sequences (figure 1). In addition to the corals, medaka fish, orangutang and human from table 1, we added Mg transporter NIPA sequences from invertebrate, amphibians, reptiles and birds, to investigate the history of Mg transporters’ evolution. The tree indicates that the Mg transporters of the Pleistocene group (*A. digitifera*, *A. hyacinthus*, *A. millepora* and *P. australiensis*) showed higher similarity to all the vertebrate Mg transporters, which is consistent with the results from table 1. These four species have both Mg transporter NIPA and NIPAL and showed the possible evolution from NIPAL to NIPA. This tree shows that vertebrate’s Mg transporters NIPA could be originated in NIPAL from the pre-Pleistocene invertebrate group.

## 3. Discussion

Remarkably, based on the available data, the Mg transporter of the coral that appeared in the Pleistocene was nearly identical to the NIPA of vertebrate (e-values <1 × 10^−40^ and sequence identity >58%). NIPA is known to be the only transporter that is highly selective for Mg^2+^; other transporters mediate a number of divalent cations [22]. NIPA helps to maintain Mg^2+^ influx to mammalian cells and has also been reported to play a critical role in osteoporosis [23]. Whether this Mg transporter is directly linked to coral skeletogenesis or is located on the membrane of the coral calicoblastic layer (skeletogenic tissue) requires experimental confirmation. However, considering that the Mg transporter sequence of *A. digitifera* is almost identical to NIPA, which is known to regulate osteoblasts [23] and was an orthologue to that in vertebrates, it is reasonable to assume that coral Mg transporter is involved in coral skeletogenesis.

Magnesium in coral skeletons has been reported to be an inaccurate proxy of past environmental parameters such as sea temperature. Our results indicated that the Mg-specific transporter of *Acropora* species has evolved over a few million years (<2.58 Ma) and is an orthologue of that in vertebrates. Based on our phylogenic analysis, *Acropora* and *Porites* species may have developed a NIPA transporter, known to be involved in osteoporosis, as the result of NIPAL evolution. The NIPA domain sequence has been preserved over a few million years and is highly similar to the human Mg transporter. This intriguing result from our sequence similarity search indicates approximately when coral speciated and began to exhibit a similar sequence to vertebrate Mg transporters. Now, the question is what could possibly stimulate the evolution of Mg transporter?

One possible scenario that could cause such a change is the availability of Mg ions in seawater (seawater [Mg]). The Mg concentration in seawater over the Cenozoic Era (65 Ma to present) has been extensively studied [24]. According to the seawater chemistry model [25], seawater [Mg] has continuously increased from 50 mmol L^−1^ at 5 Ma to 55 mmol L^−1^ at the present day. This approximately 10% increase in seawater [Mg] could potentially have stimulated coral Mg transport systems to respond to changes in seawater chemistry and evolve to develop Mg-specific transporter NIPA. Interestingly, it has been reported that lower Mg:Ca ratios in seawater result in the production of skeletons with greater amounts of calcite [26]. This finding indicates that Mg plays a significant role in the determination of aragonite/calcite skeletons regardless of whether Mg transportation is abiogenic or biogenic. It has been reported that coral skeletal formation is a result of two co-systems (particle attachment and ion-by-ion filling), and the centre of calcification includes Mg-rich organics suggesting that Mg serves as a basis for crystal growth [27]. Considering this, Mg transportation plays an important role possibly as a seed for crystal growth and could be subject to evolutional changes due to the surrounding seawater [Mg].

Another scenario that could cause the evolution of Mg transporter is that corals possibly obtained a more efficient method to regulate Mg concentrations to control skeletal growth. Mg in coral skeletons are not incorporated into aragonite lattice structures; rather they are located on the side of lattice structure based on their X-ray absorption fine structure data [28]. This indicates that Mg is part of the skeletal organic matter instead of a part of skeletal lattice structure. Also, Mg does not form Mg-rich clusters inside aragonite [29]. In addition, there may be physiological controls on Mg concentrations in aragonite-secreting organisms such as corals and bivalves [30]. These findings are consistent with the recent study that human Mg transporter regulates the Mg flux into bone cells, which impact the expression of bone formation genes by changing cAMP level ([20]; i.e. Mg concentration works as a controlling factor to regulate the bone synthesis instead of Mg themselves being part of bone structures).

In either case, it suggests the Mg transport system from seawater to coral skeletons can be biogenic, and this supports why the Mg ratio in coral skeletons is not an ideal proxy to investigate past abiogenic factors. Although other elements such as Sr are present in coral skeletons, it has been reported that no Sr-specific transporter was found in the *A. digitifera* database unlike Mg [14]. Thus, Sr is most likely carried from seawater into the skeletons abiogenically compared with Mg. This can explain why a number of studies show that coral skeletal Sr is a robust geochemical proxy compared with Mg [1,2,4,5,7,8].

Interestingly, coral skeletons have been used as both human bone void fillers and bone grafts in medical research [31]. Mg is considered an integral part of the inorganic structure of human bones and teeth [32], and approximately 60% of the Mg in the human body is stored in the skeletons [33]. It has been accepted that the coral biomineralization system is comparable to that of humans, implying that some domain sequences have been preserved for millions of years. Such evolutionary commonalities between corals and humans can be evidence of biological complexity and thus may help us to understand the magnitude of biogenic effects in ion trafficking from seawater to coral skeletons.

Investigating protein databases and conducting sequence similarity searches are useful for differentiating abiogenic from biogenic processes in marine calcifiers. In addition, reviewing species divergence data can indicate the evolutionary success of a specific domain, which is useful for evaluating the magnitude of biogenic effects. Although the experimental confirmation is still necessary to evaluate if Mg transporter is indeed involved in coral skeletal formation, this study attempted to use the results of phylogenic tree analysis to aid geochemical interpretation. Abiogenic paths should not be subject to evolution, and coral Mg transporters that possibly involved in coral skeletogenesis showed gene evolution from NIPAL to NIPA and high similarities to vertebrate Mg transporter NIPA. This approach will facilitate the identification of geochemical proxies with strong biogenic effects and provide the logical explanation to select what elements can be effective in reconstructing past ocean environments.

## 4. Material and methods

### 4.1. Coral data

Elemental compositions of *Acropora* sp. have recently received attention as a new sea temperature proxy [8]. Furthermore, *A. digitifera* is the first coral species for which the whole genome has been sequenced [34], thus the database with annotations is well established. We used three published coral databases: the Okinawa Institute of Science and Technology (OIST) genomic project *A. digitifera* v. OIST_v.1 and 2 (https://marinegenomics.oist.jp/coral/viewer/info?project_id=3; https://marinegenomics.oist.jp/adig/viewer/info?project_id=87); Reefgenomics [35] http://reefgenomics.org/); and the Coral Trait Database ([36]; https://coraltraits.org/). The OIST genomic project *A. digitifera* v. OIST_v.2 was chosen to identify the coral Mg transporter of *A. digitifera*. Protein databases in Reefgenomics were used to investigate Mg transporter genes in eight coral species (*A. digitifera*, *A. hyacinthus*, *A. millepora*, *A. palmata*, *A. tenuis, P. australiensis, P. lobata* and *P. astreoides*). Estimation of the divergences time within the phylogeny of these five species was obtained from the Coral Trait Database (section; species age phylogeny data).

### 4.2. Vertebrate and invertebrate data for comparison with coral data

To investigate the evolution of coral Mg transporters that may be related to vertebrate skeletogenesis, first we considered three species: Japanese rice fish (*O. latipes*), Sumatran orangutan (*P. abelii*), and humans (*Homo sapiens*). We chose Japanese rice fish (*O. latipes*) and Sumatran orangutan (*P. abelii*) because they were shown to have the highest protein sequence similarity to Mg transporter of *A. digitifera* based on the OIST genomic project *A. digitifera* v. OIST_v.1 and 2, respectively. Also, the comparison between corals and human is essential based on the previous study reporting sequence similarity of corals and human [18]. Next, we added one species from invertebrates (worm; *Caenorhabditis briggsae*), amphibians (frog: *Xenopus laevis*), reptile (gecko: *Heteronotial binoei*) and bird (chicken: *Gallus gallus*). Protein-sequence data for these vertebrate and invertebrate species were obtained from the NCBI protein database using the keywords as NIPA magnesium transporter (https://www.ncbi.nlm.nih.gov/protein/).

### 4.3. Sequence similarity search of Mg transporters among five *Acropora* sp. and three *Porites* sp.

We used OIST genomic project *A. digitifera* ver. OIST_v.2 to find the protein sequence of the Mg transporter and used this as the query sequence. From the Reefgenomics database, protein data of five *Acropora* and three *Porites* species were extracted as subject sequences. The numbers of proteins available in the *A. digitifera*, *A. hyacinthus*, *A. millepora*, *A. palmata*, *A. tenuis, P. australiensis, P. astreoides* and *P. lobata* datasets were varied: 16 977, 11 589, 28 463, 7522, 18 419, 19 567, 15 755 and 21 062, respectively. Using these query and subject sequences, BLAST was conducted using BLASTP 2.15.0^+^ [37,38].

### 4.4. Comparison of Mg transporters in five *Acropora*, three *Porites* sp. and humans

A total of 202 human Mg transporter sequences searched using Mg transporter as a keyword were obtained from the NCBI protein database (https://www.ncbi.nlm.nih.gov/protein/). Using them as query sequences, BLAST search was conducted to identify the human Mg transporter that had the highest similarity with five *Acropora* sp. and three *Porites* sp. from Reefgenomics database.

### 4.5. Statistical analysis (Phylogenic analysis)

A total of 20 Mg transporter sequences among different coral species and organisms discussed above were aligned using clustal omega (https://www.ebi.ac.uk/Tools/msa/clustalo) to identify locations of common sequences [39], the aligned sequences were trimmed with Clipkit to remove uninformative sequence sites and increase the accuracy of phylogenic tree ([40]; https://clipkit.genomelybio.com/#/). Finally, we built a phylogenic tree based on the neighbor joining method using Simple Phylogeny to investigate the history of Mg transporter evolution ([39]; https://www.ebi.ac.uk/jdispatcher/phylogeny/simple_phylogeny).

## Data Availability

The data and code used in this study are available in the supplement files [41]. Also, data and relevant code for this research work are stored in GitHub: https://github.com/TomoCoral/Bioinformatic-approach-to-explain-how-Mg-from-seawater-may-be-incorporated-into-coral-skeletons- and have been archived within the Zenodo repository [42].
